# Various challenges in understanding the thick filaments, within and outside skeletal and cardiac muscles

**DOI:** 10.1007/s12551-025-01289-8

**Published:** 2025-02-27

**Authors:** Jean Emile Morel

**Affiliations:** Laboratoire de Biologie at Ecole Centrale Paris, 92295 Châtenay-Malabry Cedex, France

**Keywords:** Interacting-heads motif (IHM), Electron microscopy (EM), Super-relaxed state (SRX state), Cell-associated water, Electrical charges, Aging

## Abstract

Thick filaments isolated from various sources, most frequently skeletal and cardiac muscles, have been studied, but several aspects of their behavior remain to be clarified. Myosin II is the principal component of these filaments. A “traditional” interacting-heads motif (IHM) has been observed in isolated thick filaments. In this motif, the two heads of the myosin II molecule interact and are stuck to the backbone of the filaments. Another aspect, the super-relaxed state (SRX state), has been described in situ, in relaxed demembranated muscle fibers and myofibrils. It has frequently been claimed that the IHM and the SRX state are closely related. Some authors still consider this relationship valid, but this view is now broadly called into question. These two phenomena occur in very different conditions, making it difficult to determine if and how they are related. For example, macromolecular crowding is a characteristic feature in situ (regardless of interfilament spacing), but not in the conditions in which the “traditional” IHM has been observed. Recent studies in situ have attempted to resolve this problem, but some of the reported findings conflict. Moreover, the association of other proteins with the myosin filaments in situ increases thick filament complexity. Experimental conditions may affect the results obtained but the consideration of long-overlooked data would help to prevent erroneous interpretations. For instance, neither the absence (EM studies) or presence (in situ studies) of cell-associated water nor electrical charges are taken into account in any of the published studies in this domain and the omission of these two parameters could lead to contradictory conclusions. My principal objective here is to provide a brief overview (with a limited number of illustrative references) of the increasing complexity of our understanding of thick filaments over the years, particularly as concerns the weak coupling or absence of coupling between the IHM and the SRX state (recent findings that may be difficult to interpret).

## Introduction

Many studies concerning the structure of the thick filaments and the IHM have been published (see for instance Alamo et al. 2017; Al-Khayat 2013; Lee et al. 2018; Woodhead and Craig 2020; Woodhead et al. 2005; Zoghbi et al. 2008; Wendt et al. 2001 presented a pioneering study of heavy meromyosin (HMM—two-headed subfragment) from smooth muscle, reporting results reminiscent of those for thee discovery of the IHM in thick filaments). The IHM has mostly been described on isolated single and “nude” thick filaments without associated proteins, in studies based on various EM techniques. However, Padrón et al. (2023) have shown that there are several different forms of IHM, in addition to this “traditional/canonical” form (see also Childers et al. 2024 concerning other unexpected properties of the IHM). As a result, there is currently no clear, definitive definition of the IHM. The SRX state* (Craig and Padrón 2022; Hooijman et al. 2011; Nag and Trivedi 2021; Stewart et al. 2010), in which the myosin molecules display very low rates of MgATP hydrolysis, has often been considered to be closely related to the IHM. In this state, the heads of the myosin molecules interact to generate a highly ordered arrangement. However, the SRX state has been observed in very different conditions, mostly in studies on whole demembranated fibers or myofibrils (in situ), although, Chu et al. (2021) and Gollapudi et al. (2020, 2021) have reported the observation of these phenomena in vitro**. Despite these findings, the situation remains unclear. In a preliminary study of cardiac synthetic filaments, Gollapudi et al. (2020) concluded that the IHM and the SRX state are uncoupled. Gollapudi et al. (2021) subsequently demonstrated that synthetic filaments are a powerful tool for studying SRX states, but without clearly confirming the uncoupling of the IHM and SRX states. Ge et al. (2024) also concluded that the IHM and the SRX state were uncoupled in another preliminary study of cardiac heavy meromyosin (HMM—two-headed subfragment). Mohran et al. (2024) performed new biochemical experiments on cardiac porcine HMM in vitro and raised questions about the validity of certain techniques used to define the SRX state, although the situation could be different in the sarcomere. In addition, Jani et al. (2024) performed X-ray diffraction and MgATP turnover assays on porcine myocardium and showed that there was no direct relationship between the definitions of the OFF (IHM) and ON states of myosin and those of the SRX and DRX states**.

In this brief review, I try to analyze and comment on some of the many-faceted aspects of the thick filaments studied under various conditions and with different experimental techniques. I consider only the problems raised by skeletal and cardiac muscles here and do not, therefore, focus on smooth or tarantula muscles. I also leave aside several important phenomena, such as the effect of interfilament and thick filament strain on myofibrillar activation and the effect of osmotic compression on myofilaments.

## Increasing difficulties

There are points of contention regarding the structure of the thick filaments. One of the key issues concerns macromolecular crowding, which affects all whole fibers and myofibrils, regardless of interfilament spacing (see for example Irving et al. 2000; Konhilas et al. 2002; Morel 2015 for analyses concerning the behavior of demembranated muscle fibers undergoing lateral compression—see also Bachouchi and Morel 1989), but is not observed in isolated single thick filaments. There have been many studies of this phenomenon, but those of Ellis (2001), Minton (2006) and Ge et al. (2016) are particularly relevant, suggesting possible effects on myosin and actomyosin MgATPase activity, with probable consequences for the definition of the SRX state (see also *). The results of experiments performed in vitro (see Introduction) may, therefore, be different from those obtained in situ. The importance of studying the IHM, the SRX state, and other complex phenomena in situ is highlighted by the work of Caremani et al. (2021), who studied the characteristics and properties of thick filaments experimentally in relaxed/resting mammalian skeletal muscle (demembranated fiber bundles and intact muscles), at 10 and 35 °C and with different interfilament spacing conditions. They described a more complex interfilament medium, containing titin*** and MyBP-C*** (myosin-bound protein C). Their findings strongly suggest that typical descriptions of the IHM may be an oversimplification (as stressed by Caremani et al. 2019 and Marcucci 2023—see again ***). Many studies on demembranated fibers or fiber bundles make use of material obtained by glycerination. Millman (1998) was critical of this technique, which is likely to generate erroneous results that may be inconsistent with those for material obtained by mechanical skinning or soft chemical permeabilization. This view was also supported by Bartels and Elliott (1982). Moreover, Pinset-Härström and Whalen (1979), using traditional EM, found that aged myosin stored at —20 °C in glycerin lost its ability to form synthetic filaments. Thus, depending on the duration of demembranation, the myosin in thick filaments may be at least partially denatured. Many cryo-EM and cryo-electron tomography (cryo-ET) experiments have been performed on skeletal and cardiac muscles. The results obtained in these studies may conflict with those obtained at higher temperatures with other techniques, highlighting the important effect of temperature. For example, Malinchik et al. (1997) reported that thick filaments from relaxed demembranated rabbit psoas muscle behaved very differently at 4 and 20 °C. This phenomenon is almost certainly related to the crossbridges in natural filaments from rabbit being ordered at room temperature and disordered at 4 °C (Kensler et al. 1994—results obtained by EM) rather than the dependence of MgATPase activity on temperature. Cianfrocco and Kellogg (2020), Hylton and Swulius (2021), Lau et al. (2022) and Lyumkis (2019) have also discussed the positive and some negative aspects of cryo-EM and cryo-ET techniques.

## Thick filaments in the sarcoplasmic medium

The sarcoplasm contains “cell-associated water”, which differs from bulk water (Drost-Hansen and Clegg 1979; Pollack 2003, 2013; Wiggins 2008; see also Grazi 2008, Morel 1985, 2015; Oplatka 1994 regarding water in muscle) and may affect the structure and function of thick filaments. In situ, thick filaments are bathed in an aqueous medium at a pH of about 7 and they carry many negative fixed charges and bound anions (Aldoroty et al. 1985, 1987; Bartels and Elliott 1980; Elliott 1980; Morel 2015). By contrast, EM techniques (other than those used by Sugi et al. 2015) generally involve the observation of thick filaments in the dry state, with no net charge. These differences would affect the behavior of the filaments, potentially leading to erroneous interpretations. The head-head and head-backbone interactions observed in situ may, indeed, be different from those observed by EM.

## Complexity and forgotten results

In this complex context, Wang et al. (2021) used cutting-edge techniques to study mouse psoas sarcomeres in situ (i.e. in favorable conditions) and in rigor, in the absence of MgATP. They obtained promising results for myosin — including information about the positioning of the two heads — and actin, and the assembly of these proteins into thick and thin filaments, respectively. They concluded that further studies of filaments in a relaxed state (in the presence of MgATP) were required and such work has now been published (Tamborrini et al. 2023, and conclusion). Koubassova et al. (2022) tried to reconcile the results of EM and X-ray diffraction experiments. However, the complexity of the interfilament medium renders the interpretation of their findings less than straightforward and their conclusions are at odds with those of Knupp et al. (2019). The complexity of the sarcoplasmic and interfilament media is a weakness common to both these studies, but several specialists have suggested that the approach of Koubassova et al. (2022) is more appropriate. Another factor that can affect the results obtained is the age of the animal from which the muscles are obtained, as shown by Morel et al. (1999), in an experimental study on synthetic and natural myosin thick filaments from male rabbits performed in vitro (for example, MgATPase activity is lower for natural filaments from old rabbits than for those from young adult rabbits). Phung et al. (2018) have also reported effects of the age of male and female mice on SRX states. Craig and Padrón (2022), Nag and Trivedi (2021), and Walklate et al. (2022), through descriptions of the disordered relaxed state (DRX state, assumed to be in reversible equilibrium with the SRX state) and many other characteristics, have also revealed a much greater complexity than was anticipated (see "Thick filaments in the sarcoplasmic medium"). Spudich et al. (2024 and references therein) recently presented a careful and complex analysis of the relationship between hypertrophic cardiomyopathy (HCM), the IHM and the SRX state under various conditions. This many-faceted complexity may, again, affect the results obtained.

## From relaxation to contraction — some recent and updated studies

Another problem that remains to be resolved relates to the mechanism of transition from relaxation/rest to active contraction (isometric and isotonic). Most studies on the IHM and the SRX state have focused on the thick filaments in a state of relaxation, with little or no consideration of contraction. Only the notions of the ON and DRX states relate to the transition between relaxation and contraction. These shortcomings are addressed briefly here. Brunello et al. (2023), Brunello and Fusi (2024) and Marcucci (2023) have suggested a plausible approach for addressing this question. They do not dismiss the traditional role of myosin motors, but instead focus on the intricate interactions between myosin and actin, taking into account the roles of the thick and thin filaments. In their well-documented analysis, Rassier and Månsson (2025) deal with many long-known and more recently identified features of contraction, taking into account the SRX-DRX states. In a monograph considering both relaxation/rest and active contraction, Morel (2015) revisited the existing literature and described unpublished experiments demonstrating a key role for thick filaments in a hybrid model of contraction/relaxation according to which contraction results from a combination of the actions of the swinging crossbridges (lever-arm mechanism) and lateral forces exerted by the thick filaments.

## Conclusion

So, several issues remain unresolved and there are controversies as to the nature of the IHM and the SRX state and the relationship between them. The resolution of these issues is complicated by the complexity of the interfilament environment and the non-negligible effects of experimental conditions on the results obtained. Additional studies are required to address the questions raised here. Several very interesting experiments based on cryo-EM and cryo-ET have recently been published, confirming the existence of the IHM and the fine structure of the thick filaments and the proteins associated with them, principally titin*** and MyBP-C*** (Dutta et al. 2023; Grinzato et al. 2023). Tamborrini et al. (2023—see also Al-Khayat 2013; Woodhead et al. 2005; Zoghbi et al. 2008) seem to have overcome the problem of macromolecular crowding highlighted in "Increasing difficulties" and that of the complexity of the sarcoplasmic medium (see "Thick filaments in the sarcoplasmic medium and Complexity and forgotten results") by studying the structure of the thick filaments in situ (relaxed myofibrils; see also Walklate et al. 2022 concerning the SRX state in relaxed myofibrils). Other studies are sure to follow, but certain precautions will need to be taken, as outlined here, to ensure the correct interpretation of the results (for example, the age of the animals studied and the temperature at which the experiments are performed should be taken into account). Furthermore, future studies may extend to isometrically contracting demembranated fibers or myofibrils, in which case other techniques would be required to observe the thick filaments and their behavior either in vitro or in situ, in the presence of MgATP and calcium (preferably at room temperature for humans and warm-blooded animals). Woodhead and Craig (2020) have studied the effect of the electrical charges on myosin II on the IHM, and Gollapudi et al. (2021) have investigated the electrostatic forces in S1 (single head), HMM and synthetic filaments. The roles of the electrical charges and of cell-associated water should always be taken into account, both at rest and in contraction conditions, and the role of the crossbridges weakly bound to actin remains to be clarified****.

## Notes

* In the SRX state, MgATPase activity is generally considered to be an order of magnitude lower than that in solution. However, the true level of activity remains unclear because the maximal relative margin of error on MgATPase activity in situ (myofibrils) lies between 1 and 8 (Morel 2015, Section 6.3.2). Another question has been raised by Phung et al. (2020) concerning the dependence of the SRX state on fiber type in human skeletal muscle. Comparisons of MgATPase activity in solution (bulk/free water) and in situ are risky due also to the presence of cell-associated water in the sarcoplasmic medium (see "Thick filaments in the sarcoplasmic medium"). In any event, a clear definition of the “resting” MgATPase activity in vitro is required before any firm conclusions can be dawn regarding the SRX states(s).

** In an experimental study on synthetic filaments, Morel et al. (1999) showed that the filaments interact strongly via the extruding heads (S1-S1 dimerization demonstrated in several papers by my group) in the presence of MgATP (relaxing conditions). This phenomenon almost certainly occurs in HMM (we found that entire myosin molecules form head-head dimers in the presence of MgATP). Caution is therefore required in the interpretation of in vitro experiments (HMM, myosin, synthetic filaments). Information about S1 and myosin dimerization can be found in Morel et al. (1998a, b and references therein).

*** The elongated giant protein titin (MW ~ 4 MDa) is tightly associated with the outer surface of the thick filaments and is located between the Z disks and the M lines. It therefore seems likely that the IHM would be at least partly hindered by titin and possibly also by MyBP-C (MW ~ 140 kDa).

**** To my knowledge, in most studies on whole muscles, demembranated fibers and myofibrils analyzed briefly here, the crossbridges weakly bound to actin (see for example Chalovich and Eisenberg 1982; Kraft et al. 1995; Xu et al. 1997) are not taken into account. Fortunately, Ma et al. (2023) included this class of crossbridges in their study of porcine myocardium.

## Data Availability

No datasets were generated or analysed during the current study.
